# NAFLD, Estrogens, and Physical Exercise: The Animal Model

**DOI:** 10.1155/2012/914938

**Published:** 2011-08-10

**Authors:** Jean-Marc Lavoie, Abdolnaser Pighon

**Affiliations:** Department of Kinesiology, University of Montreal, Montréal, QC, Canada H3C 3J7

## Abstract

One segment of the population that is particularly inclined to liver fat accumulation is postmenopausal women. Although nonalcoholic hepatic steatosis is more common in men than in women, after menopause there is a reversal in gender distribution. At the present time, weight loss and exercise are regarded as first line treatments for NAFLD in postmenopausal women, as it is the case for the management of metabolic syndrome. In recent years, there has been substantial evidence coming mostly from the use of the animal model, that indeed estrogens withdrawal is associated with modifications of molecular markers favouring the activity of metabolic pathways ultimately leading to liver fat accumulation. In addition, the use of the animal model has provided physiological and molecular evidence that exercise training provides estrogens-like protective effects on liver fat accumulation and its consequences. The purpose of the present paper is to present information relative to the development of a state of NAFLD resulting from the absence of estrogens and the role of exercise training, emphasizing on the contribution of the animal model on these issues.

## 1. Introduction

Liver is particularly vulnerable to ectopic fat accumulation that results in NAFLD characterized by hepatic lipid accumulation (5 to 10% per weight) in the absence of significant alcohol consumption [1]. In recent years, there has been increasing evidence that NAFLD by itself has important metabolic implications. Some authors refer to NAFLD as “insulin resistance associated steatosis” since all components of the metabolic syndrome correlate with liver fat accumulation independently of obesity [2]. NAFLD is becoming a risk factor for diabetes and cardiovascular diseases (CVDs) independently of insulin resistance, metabolic syndrome, plasma lipid levels, and other usual risk factors [3, 4]. NAFLD has also been shown to predict both type 2 diabetes and CVD independent of obesity. In addition, hepatic steatosis by itself is associated with a proatherogenic lipid profile and increased production of proinflammatory markers [5, 6].

The general association between NAFLD and CVD was established by the fact that the liver is involved in regulating/secreting numerous CVD risk factors, notably the cytokine tumor necrosis factor-alpha (TNF-*α*), an acute-phase protein CRP, glucose, lipoproteins, coagulation factors (plasminogen activator inhibitor-1), and a substance which increases blood pressure (angiotensin II) [7]. These authors claim that “hepatocytes are the last cells to be involved in the progressive chain of fat accumulation and probably the first cells to tell us that something is wrong”.

The exact pathogenesis of hepatic lipid accumulation seems to be very complex and only partially understood. As a whole, the general mechanism of liver fat accumulation involves an imbalance between lipid availability (from circulating lipid uptake or *de novo* lipogenesis) and lipid disposal (through fat oxidation or triglyceride-rich lipoprotein secretion) [8, 9]. Excessive fat accumulation in liver can occur as a result of increased fat delivery into the liver (dietary fatty acids and plasma nonesterified fatty acids derived from adipose tissue), increased fat synthesis in liver, reduced fat oxidation, and reduced fat exportation in the form of VLDL. Considering the complexity and heterogeneity of the mechanisms involved, it is quite difficult to imagine that it would be possible to identify a single gene variation as the single cause of the disease [10]. Body fat, insulin resistance, oxidative stress, mitochondrial dysfunction, cytokine/adipokine interplay, and apoptosis are potential risk factors of NAFLD [10]. 

One segment of the population particularly inclined to increased hepatic fat accumulation is postmenopausal women. Two thirds of postmenopausal women are considered overweight or obese and 43% present the metabolic syndrome [11]. Recent evidence indicates that menopause is indeed associated with the development of a state of hepatic steatosis [12]. Population-based studies indicate that nonalcoholic hepatic steatosis is more common in men than in women. However, following menopause there is a reversal in gender distribution so that NAFLD is more common in women than in men [13]. In fact, it has been reported that nonalcoholic liver steatosis is twice as common in postmenopausal compared to premenopausal women, and that hormonal replacement therapy decreases the risk of steatosis [14]. Basic and clinical studies do support the hypothesis that estrogens protect from the development of NAFLD [15, 16]. In addition, alterations in body composition, fat distribution, and/or hormonal or metabolic changes that occur following menopause may influence the development and progression of NAFLD [17].

It seems that hormone replacement therapy decreases the risk of steatosis [14] as the prevalence of NAFLD is lower in postmenopausal women taking hormone replacement therapy than in women not taking it [18]. Nevertheless, although hormone replacement therapy appears safe in NAFLD, it is not recommended for liver protection because of the increased risk of cardiovascular events [19, 20]. In a recent review on NAFLD in older women, it was concluded that at present, there are no specific or effective pharmacological treatments available, and lifestyle modifications with weight loss and exercise are regarded as first line treatments [21].

The purpose of the present review is to present information relative to the development of a state of hepatic steatosis with estrogens withdrawal and the role of physical exercise to circumvent this phenomenon, emphasizing the contribution of the animal model on these issues.

## 2. Estrogens Withdrawal in Animals: Central and Peripheral Effects

### 2.1. Central (Extra-Hepatic) Effects of Estrogens Withdrawal

Ovariectomy (Ovx) in animals leads to increased food intake and body weight along with increased adipose tissue and liver fat accretion [22–24]. Data from observational and clinical trials support the fact that estrogens possess favourable metabolic effects as estrogens treatment has been shown to decrease body weight gain and fat accumulation in both animals and humans [25, 26]. In addition to increased food intake there is some evidence that energy expenditure is decreased with estrogens deficiency. A 40% reduction in ambulatory activity levels has been reported after ovariectomy in mice [27]. There are, therefore, central effects of estrogens withdrawal that are responsible for increased food intake and decreased energy expenditure resulting in adipocyte fat gain preferably in intra-abdominal region. 

Since hyperphagia is a well-known response to Ovx and is prevented by estradiol replacement, many of the effects attributed to estradiol may be explained primarily by changes in food intake [28]. In fact, one view of Ovx-induced obesity in rat is that estrogens removal leads to a marked increase in body energy stores via increased energy intake and food efficiency along with decreased energy expenditure, which leads to increased energetic efficiency [24, 29]. This contributes to weight gain, especially as visceral or intra-abdominal fat, as observed in Ovx animals [30] and in women during and after menopause [31, 32]. Consequently, determinants of lipid metabolism such as liver triacylglycerol level and adipose tissue lipoprotein lipase (LPL) activity are altered in correspondence with an increased energy flux [29]. In other words, Ovx-induced increased energy efficiency is accompanied by concomitant adaptations of peripheral lipid metabolism that include the induction of pathways implicated in fat accumulation [23]. Therefore, the central effects of estrogens withdrawal on food intake and changes in insulin levels and its efficiency of action may indirectly affect liver fat accumulation in Ovx animals [24]. Central effects of estrogens supplementation in Ovx rats have been shown to lower food intake [33, 34], decrease adipose tissue LPL activity [35], and increase adipose tissue lipolysis [36], spontaneous physical activity [37], and energy expenditure [34, 38]. With regard to the central effects of estrogens, Picard et al. [24] postulated that Ovx induces obesity by removing the catabolic actions of estrogens, which act upon, yet poorly defined, central neuropeptidergic pathways that regulate energy balance. On the whole, there is little doubt that estrogens exert central effects that regulate feeding and energy expenditure through direct actions on the hypothalamus and/or through indirect actions by regulating adipose hormones such as leptin, adiponectin, and resistin [39].

### 2.2. Peripheral (Intrahepatic Effects) of Estrogens Withdrawal: Molecular Implications

In addition to the central effects, it is now well recognized that almost all tissues are under estrogenic influence in both men and women [40, 41]. Epidemiological and clinical evidence strongly suggest that estrogens, in particular 17 *β*-estradiol, the most potent and dominant estrogens in mammals, play an important regulatory role in the metabolism and regional distribution of adipose tissue [42–44]. Estrogens promote subcutaneous fat depot after sexual maturation [43], while estrogens deficiency leads to increased fat, predominantly in visceral tissue [45]. It seems that estrogens control fat distribution by changing the lipolytic response distinctly into the two fat deposits, thus favouring fat accumulation in peripheral depots at the expense of the visceral depot [45]. There is also evidence that estrogens regulate LPL activity. It has been shown in several studies that ovariectomy in female rats results in increased adipose tissue LPL, while estrogens replacement decreased LPL activity [44].

In recent years, it has become evident that estrogens' role in adipose tissue biology and lipid metabolism may be broader and more complex than initially appreciated. It seems that active metabolic tissues, such as the liver, are particularly sensible to estrogens effects in terms of different functions including lipid metabolism. The molecular and biological mechanisms underlying the metabolic actions of estrogens in liver are weakly understood. Estrogens are a steroid hormone mainly produced by ovaries whose actions are predominantly mediated by genomic mechanisms through its nuclear receptors (ER) *α* or *β* [46]. Outstanding advancements in recent years indicate that estrogens action in vivo is complex and often involves activation of cytoplasmic signalling cascades in addition to genomic actions mediated directly through estrogens receptors *α* and *β*. Estrogens may simultaneously activate distinct signalling cascades that function as networks to coordinate tissue responses [47]. These orchestrating distinct signalling pathways which involve specific complexes of cytoplasmic proteins might supplement or augment genomic effects of estrogens that are attributable to transcriptional activation by liganded receptors [48]. Therefore, it is not surprising that estrogens have been shown to exert rapid non-genomic biological actions through membrane bound subpopulations of ERs [49–51]. Interestingly, D'Eon et al. [52] reported novel genomic and non-genomic actions of estrogens that promote leanness in Ovx animals independently of reduced energy intake. In a recent review on estrogens regulation of adipose tissue functions, it was reported that estrogens reduce adiposity by promoting the use of lipid as fuel which is recognized by the activation of pathways that promote fat oxidation in muscle, by inhibition of lipogenesis in adipose tissue, liver, and muscle and by improved rates of adipocyte lipolysis [45]. The precise mechanism by which estrogens affect these functions is still unknown.

**Figure 1:**
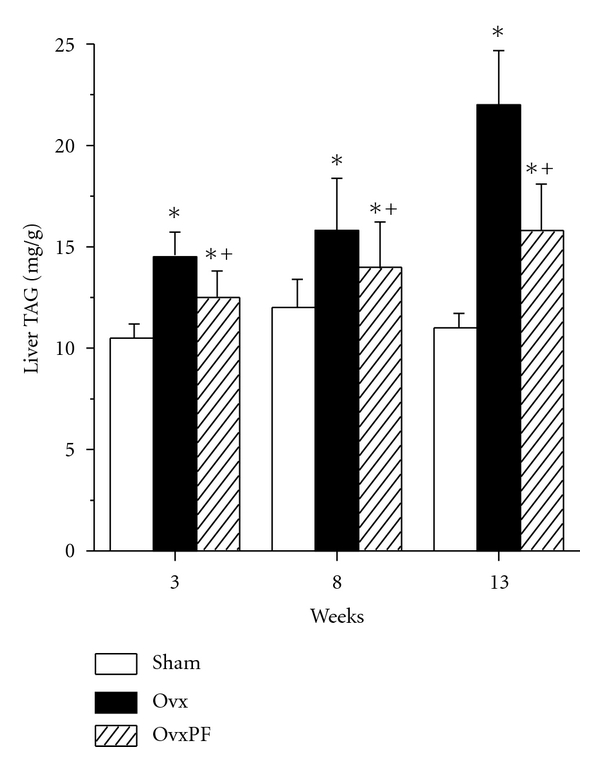
Effect of energy restriction in Ovx rats to the same level as food intake in intact rats on the hepatic TAG accumulation. *n* = 8/group. Sham: sham operated; Ovx: ovariectomized; OvxPF: Ovx rats paired fed to the level of Sham rats. *Significantly different from Sham *P* < 0.05; + Significantly different from Ovx *P* < 0.05. Taken in part from Paquette et al. [30].

Estrogens-deficient state in ovariectomized animals has been repeatedly shown to result in a rapid liver fat accumulation [30, 53, 54]. Hepatic steatosis also developed in aromatase-deficient mice (ArKO; lacking the intrinsic ability to produce estrogens) and is diminished after treatment with estradiol [55]. Visceral obesity, metabolic syndrome with insulin resistance, as well as hepatic steatosis are the main features of the ArKO's mouse phenotype [56]. Although many of the effects attributed to estrogens in the pathogenesis of Ovx-induced fat gain may be explained to a certain extent by the central effects of estrogens mostly via changes in food intake, D'Eon et al. demonstrated that estrogens reduced adiposity in Ovx rodents without confounding differences in food intake [52]. Their data are consistent with the phenotypes of both estrogens receptors-*α* (ERKO) knock-out and ArKO mice, both of which exhibit increased adiposity with no reported differences in food intake [38, 57–59]. Moreover, the results of Beckett et al. [60] suggest that estradiol regulates substrate metabolism in ectopic tissues such as skeletal muscles independently of changes in food intake. On the whole, it becomes evident that the ovarian hormonal status has important ectopic effects at the molecular level in peripheral tissues such as the liver rather than only central effects on food intake and energy expenditure. Accordingly, Fisher et al. [61] reported that despite a similar food intake, Ovx-pair fed animals gained markedly more weight than did Sham animals and nearly as much as Ovx-*ad libitum* animals. Likewise, data collected from our lab indicate that pair-feeding in Ovx rats does not completely prevent liver fat accretion in rats (Figure 1). Therefore, there must be factors other than food intake in the pathogenesis of liver fat accumulation in estrogenic-deficient state. 

Some intrahepatic pathways leading to lipid infiltration in estrogens deprived states have been investigated. Increased lipid uptake by liver as a result of increased fatty acid flow from circulation coming from intra-abdominal fat deposition attributed to the increased food intake after estrogens withdrawal can primarily and partially explain hepatic fat accumulation. The portal/fatty acid flux theory suggests that visceral fat, via its unique location and enhanced lipolytic activity, releases toxic-free fatty acids, which are delivered in high concentrations directly to the liver [62]. However, the portal/fatty acid flux theory has been questioned with the observation that the bulk of portal vein free fatty acids originate from subcutaneous adipose tissue in overnight-fasted obese individuals [63]. Nevertheless, there is little doubt that an increased arrival of lipids in situations of increased food intake and/or increased lipolysis contributes to liver lipid infiltration.

Disturbed regulatory mechanisms of lipids in the liver resulting from estrogens deficiency have been reported to play a role in liver fat accumulation. As estrogens levels decline, there may be increased lipogenesis and reduced fatty acid oxidation within the liver [17]. Liver *de novo* fatty acid synthesis that may result in hepatic steatosis is mostly regulated by three known transcription factors: SREBP-1c, ChREBP, and PPAR-*γ* [64–66]. SREBP1-c activates fatty acid synthase (FAS) and stearoyl-CoA desaturase-1 (SCD-1) genes that are responsible for lipogenesis in liver [64]. D'Eon et al. [52] investigated the expression of several genes involved in the regulation of lipogenesis in the liver of Ovx and Ovx with estrogens replacement mice. Similar to their observations in adipose tissue, estrogens supplementation in Ovx rats decreased hepatic expression of the lipogenic gene SREBP-1c and its downstream targets ACC-1 and FAS compared to Ovx control rats. Similarly, increased lipogenesis in liver of Ovx rats was supported by changes in the expression and protein content of the lipogenic markers SREBP-1c and SCD-1 [67]. Furthermore, Na et al. [68] reported that estrogens deficiency in high-fat fed rats increased liver mRNA expressions of FAS and PPAR-*γ* while decreasing mRNA levels of the oxidative marker PPAR-*α*. In line with these reports, Ovx mice displayed visible steatosis even in a state of pair-feeding that was coincident with a remarkable elevation in hepatic PPAR-*γ* gene expression and downstream target genes FAS and ACC [68]. To confirm the role of estrogens in regulation of hepatic lipid metabolism, it has been shown that 17-beta-estradiol replacement in an animal model completely prevented the accumulation of lipids in the liver of Ovx rats and normalized the disturbed lipogenesis and lipid oxidation in liver [67, 69]. In addition to increased lipogenesis, a decrease in hepatic gene expressions involved in lipid oxidation, such as PPAR-*α*, was also reported in Ovx rats. PPAR-*α* is a receptor for peroxisome proliferators that functions as a lipid sensor that, when ineffective, can lead to reduced energy burning resulting in hepatic steatosis [70]. A decrease in PPAR-*α* in liver of Ovx rats has been reported [67]. Similar findings have also been reported in liver of ARKO mice [71]. Finally, Paquette et al. [72], using a physiological approach, reported a decrease of 34% in the rate of fatty acid oxidation in isolated hepatocytes of Ovx rats. 

Besides lipogenesis and lipid oxidation, VLDL-TG production and secretion under low estrogenic condition might also be affected. In a study by Lemieux et al. [73] conducted on female Sprague-Dawley rats treated with the estrogens antagonist acolbifene, it was found that VLDL-TG secretion rate and microsomal transfer protein (MTP) mRNA levels were decreased by ~25–29%. Very recent data from our laboratory also revealed a decline in VLDL-TG production and MTP mRNA and protein content in Ovx rats [53]. Taken together, it is clear that estrogens withdrawal can have direct effects on hepatocytes and cellular constituents of liver tissue (intrahepatic effects) as well as central effects on food consumption, energy expenditure, and adipose tissue fat deposition that jointly contribute to the overall effects of liver fat accretion (Table 1).

## 3. NAFLD in Polycystic Ovary Syndrome (PCOS) Women

As mentioned above, premenopausal women are protected from the occurrence of CVD and NAFLD [16]. However, following menopause there is a reversal in gender distribution so that NAFLD becomes more common in women than in men [13]. It seems that a normal balance between androgens/estrogens ratio is required to maintain a proper distribution of body fat and normal metabolism in men and women [74]. For instance, hypoestrogenism in male rats and men is associated with fatty liver and features of the metabolic syndrome [16]. Similarly, hyperandrogenism in women is associated with increased central adiposity, insulin resistance, and increased risk of NAFLD [74]. Women with PCOS are at increased risk of metabolic syndrome and other complications such as type 2 diabetes and NAFLD [75, 76]. According to the European Society for Human Reproduction (ESHRE) and the American Society of Reproductive Medicine (ASRM), a diagnosis of PCOS requires two of the following three criteria: the presence of oligoovulation or anovulation, biochemical or clinical signs of hyperandrogenism, and the presence of polycystic ovaries [77]. Gambarin-Gelwan et al. [78] reported the presence of fatty liver in 55% of patients with PCOS, and nearly 40% of the patients diagnosed with NAFLD were lean. The beneficial effect of weight loss and exercise on liver fat accumulation of PCOS patients has been observed in a case report [79].

## 4. Exercise/Diet Interventions in Postmenopausal Women

More than 60% of American postmenopausal women are overweight or obese [80] and as mentioned earlier, it is well established that menopause is associated with weight gain, unfavourable alterations in body composition (elevated visceral fat deposition), and a state of hepatic steatosis [12, 81]. It seems that hormone replacement therapy (HRT) alleviates the metabolic consequences of menopause [82–84]. However, research on the safety of HRT is conflicting. The Women's Health Initiative in the United States in 2002 and the Million Women Study in the UK in 2003 reported evidence of increased risk of heart disease, stroke, venous thromboembolism, and breast cancer with HRT in postmenopausal women [85, 86]. In general, although short-term use of HRT remains beneficial for severe menopausal symptoms, the uncertainty with the risks/benefits of HRT along with the well-publicized results of the above two large-scale HRT trials, has led to the conclusion that HRT will not protect future health in postmenopausal women [87]. Therefore, women continue to seek alternative options to improve their quality of life and reduce the risk of heart disease, osteoporosis, and breast cancer during post-menopause time [88]. 

Interestingly, the most research recommended cornerstone prevention/treatment for weight gain, elevated visceral fat deposition, and hepatic steatosis is weight loss through lifestyle interventions including exercise and/or diet. In a recent review, Zanesco and Zaros [89] reported that in an attempt to reduce the incidence of CVD in postmenopausal women, a variety of approaches has been used with conflicting results. Nevertheless, the change in lifestyle has been proposed as the most effective preventive action. This conclusion confirms the important role played by exercise and nutrition in the prevention and treatment of obesity, diabetes, and CVD in postmenopausal women [90, 91]. Data from a 5-year randomized clinical trial known as the Women's Healthy Lifestyle Project had previously demonstrated that weight gain and increased waist circumference during the peri- to postmenopausal period can be prevented by a long-term lifestyle dietary and physical activity intervention [92].

One of the most important components of lifestyle is physical activity which has been known for a long time to be a powerful low-risk mean for the promotion of all aspects of human health including menopause [93]. Postmenopausal women might demonstrate a greater response to exercise since it was shown that even small increases in physical activity and exercise at the time of menopause can help prevent the atherogenic changes in lipid profiles and the weight gain experienced by these women [91]. Longitudinal and cross-sectional studies have shown that physical activities, such as moderate-intensity sports/recreational activity or biking and walking for transportation are associated with lower body fat and less central adiposity in postmenopausal women [81, 94, 95]. Moderate-intensity exercise (walking or 45 min moderate-intensity aerobic activity 5 d/wk) can also result in improvements in coronary/metabolic risk factors such as insulin resistance in postmenopausal women [96–98]. The results of a study by Hagberg et al. [99] even indicated that numerous years of high-intensity endurance training had a greater effect on total and regional body fat values than HRT in postmenopausal women. Given that obesity is extremely prevalent and difficult to treat, prevention of weight gain after menopause is an important health target. A successful model of weight gain prevention has yet to be established [100]. In a longitudinal study, Hagmar et al. [101] reported that former elite but still active endurance female athletes had higher flow-mediated vasodilatation, used as an indicator of endothelial function, than control subjects. This latter study does not, however, discriminate the previous exercise training conducted during the reproductive period from the training conducted during menopause. A response to this question may be tentatively obtained from a recent study in Ovx animals in which it was found that to be effective in reducing adipocytes and liver fat accumulation, exercise must be conducted concurrently with estrogens withdrawal [102]. On the whole, it seems that postmenopausal women with high levels of physical activity have lower body and abdominal fat and are less likely to gain fat (total and abdominal) during menopause than those with lower levels of physical activity [81]. Endurance training has been reported to be very effective in reducing intrahepatic triglycerides content in human (for a recent review see [103]). More recently, a 12-month intensive lifestyle intervention in patients with type 2 diabetes has been reported to reduce hepatic steatosis by as much as 25% [104]. However, information is lacking on the role of exercise training specifically on prevention and/or reversal of hepatic steatosis in postmenopausal women.

## 5. Exercise in the Animal Model of Menopause

Ovx animals can benefit from an exercise training program with a reduction in fat gain [105]. In 2002, Shinoda et al. [106] showed that exercise training exerts a strong action upon reduction in body fat accumulation following a decrease in estrogens levels. In spite of the reduction in body fat, 8 wk of endurance exercise training in this study did not reduce overall weight gain suggesting a compensatory increase in muscle weight by training. This is an interesting asset of exercise since food restriction protocols in Ovx rats have been known to be associated with a decrease in muscle mass [107]. In this regard, it was shown that muscle tissue hypertrophy induced by a progressive loading exercise program has a stimulatory effect on bone mass in Ovx rats [108]. 

A major concern of a reduction in estrogenic status is insulin resistance. It is well known that environmental factors such as aging, obesity, and physical inactivity are linked to the development of a state of insulin resistance and type 2 diabetes mellitus. Rationally, the prevalence and progression of type 2 diabetes are likely to increase in postmenopausal women. Several studies reported insulin resistance in experimental animals after Ovx, which can be reversed by HRT and exercise training although the results have been somewhat conflicting [22, 109, 110]. One of the best evidence of the effects of exercise training on estrogens withdrawal-induced insulin resistance comes from the study of Saengsirisuwan et al. [111]. These authors showed that ovariectomy in female Sprague-Dawley rats resulted in the development of a systemic metabolic condition presenting the characteristics of the metabolic syndrome including increased visceral fat content, abnormal serum lipid profile, impaired glucose tolerance, and defective insulin-mediated skeletal muscle glucose transport. Saengsirisuwan et al. [111] also provided evidence that whole-body and skeletal muscle insulin resistance is effectively corrected by endurance exercise training alone and estrogens replacement alone. Despite this and similarly to Choi et al. [112], they could not find evidence that exercise training additively modulates insulin action in Ovx animals that also received estrogens replacement, suggesting that endurance exercise training and estrogens may share common mechanisms to correct defects in ectopic tissues caused by estrogens deficiency. This concept is supported by the observations that transcripts encoding estrogens signalling in skeletal muscle, cardiac muscle, and liver are enhanced by regular exercise [113–115]. 

As previously mentioned, menopause is associated with the development of a state of hepatic steatosis [12, 116], which plays an important role in the development of insulin resistance [117]. Ectopic fat in liver may be even more important than visceral fat in the characterization of metabolic obesity in humans [118, 119]. An alternative to counteract liver fat accumulation with estrogens withdrawal may be exercise training. It has been reported that exercise training prevents fat accumulation in livers of high-fat fed rats [120, 121]. Recently, we reported evidence that endurance exercise training conducted concurrently with estrogens withdrawal did prevent liver fat accumulation in rats [102]. This latter study is particularly interesting since it also showed that if exercise is conducted only before the ovariectomy, there was no protective effect of exercise on subsequent Ovx-induced liver fat accumulation. On the other hand, if exercise was started at the same time as Ovx was performed, liver fat accumulation was prevented emphasizing the finding that exercise must be conducted concurrently as estrogens withdrawal to be effective. To explore mechanisms by which exercise prevents liver fat accumulation in Ovx rats, Pighon et al. [122] conducted a subsequent study in which they measured the expression of several genes in liver. They found that exercise training acts as estrogen supplementation in properly decreasing several genes of lipogenesis (SREBP-1c, ChREBP, SCD-1, ACC) as well as decreasing several biomarkers of subclinical inflammation (IL-6, NFkB, TNF*α*) in Ovx rats.

## 6. Resistance Training (RT) in Postmenopausal Women

Weight loss achieved through restrictive diets often results in negative effects on muscle mass [123]. In this regard, resistance training seems to be a logical choice considering its beneficial effects on muscular strength in postmenopausal women [124]. It has been demonstrated that RT exercise can be an effective substitute for hormone replacement therapy in preventing menopause-related osteoporosis and sarcopenia [125]. In addition to increasing muscle mass and improving muscle function, RT has been reported to induce decreases in total and abdominal fat [126, 127]. On the other hand, there are studies that showed no reduction in fat tissue with RT exercise [128]. Eight weeks of low intensity, short duration RT program was not sufficient to produce significant modifications in body composition and blood lipid concentrations in postmenopausal women, although it produced substantial improvements in muscle strength [129]. In obese sedentary postmenopausal women, it has been suggested that RT has the potential to ameliorate/prevent the development of insulin resistance and may reduce the risk of glucose intolerance and non-insulin-dependent diabetes mellitus [130]. In these subjects, RT alone or in combination with a weight loss program (diet) (RT+WL) improved muscular strength and insulin action and glucose homeostasis. However, the same authors in a subsequent study showed that body weight and fat mass did not change with RT alone, but decreased with RT+WL [131]. Nevertheless, RT and RT+WL both increased fat-free mass and resting metabolic rate in postmenopausal women [132]. Considering the fact that subjects in RT group were nonobese and subjects in RT+WL group were obese postmenopausal women, the authors suggested that RT may be a valuable component of an integrated weight management program in postmenopausal women. In a recent study conducted in overweight and obese postmenopausal women, it was reported than RT combined to caloric restriction was more effective that caloric restriction alone in reducing fat mass (%) and trunk fat mass [133]. Although, as for endurance training, there is a paucity of information on the impact of RT on NAFLD in postmenopausal, it seems that RT constitutes an asset to overcome several of the deleterious metabolic effects associated with menopause.

## 7. Resistance Training (RT) in Ovx Animals

In Ovx rats, Corriveau et al. found that an 8 wk program of resistance training in conjunction with restrictive diet reduced intra-abdominal fat depot and plasma-free fatty acid levels and prevented liver fat accumulation [54]. It was concluded that RT is an asset to minimize the deleterious effects of ovarian hormone withdrawal on abdominal fat and liver lipid accumulation in Ovx rats. Leite et al. [134] also recently indicated the potential benefits of resistance training as an alternative strategy to control the negative effects of ovariectomy. Twelve weeks of strength training in Ovx rats decreased fat content in liver, skeletal muscle, and intra-abdominal adipose tissue and positively changed lipid profile such as increasing HDL levels while decreasing total cholesterol and LDL levels. In both of these studies, the RT program consisted of climbing a vertical grill with weights attached to the tail of rat. 

Using the same design as Corriveau et al. [54], Pighon et al. conducted two studies on liver and body fat regain in Ovx rats using resistance training. In a first study [135], they tested the hypothesis that substituting food restriction by resistance training after a period of weight loss would maintain the decrease in fat accumulation in liver and adipose tissue that occurs with weight loss. We found that cessation of an 8 wk food restriction regimen aimed at lower body weight and fat accumulation in Ovx rats may be substituted by a resistance training program (over 5 more weeks), without causing any appreciable regain of fat in liver and adipose tissue. Our group suggested that changing from a food restriction regimen to a resistance training program may be an interesting strategy to promote successful long-term weight reduction in postmenopausal women. In a second study, Pighon et al. [136] investigated the effects of maintaining RT or food restriction on body weight regain, fat mass, and liver lipid infiltration in Ovx animals previously submitted to a food restriction + RT weight loss program. We observed that maintaining only food restriction was the most effective but that maintaining RT alone was an asset to attenuate intra-abdominal and liver fat reincrease. Again we suggested that the maintenance of only one component of an RT+ food restriction weight loss program constitutes a positive strategy to reduce body weight and fat mass relapse in postmenopausal women.

## 8. Exercise and Weight Regain

It seems that maintenance of weight loss is a core problem in the treatment of obesity, and long-term maintenance of weight loss remains a challenge. A common treatment for weight loss is food restriction or hypocaloric diet therapy. Although interventions aimed at weight loss are well supported [137], reductions in weight by dietary restriction are typically modest and are increasingly viewed as an unsustainable outcome of lifestyle modification [138, 139]. It thus seems that there is a high rate of recidivism after diet-induced weight loss. One of the main underlying problems in this matter appears to be the compensatory metabolic responses to weight reduction which results in a strong drive to regain lost weight [140, 141]. Such responses include enhanced metabolic efficiency with a progressively increasing appetite along with interrelated alterations like improved insulin sensitivity and energetically favourable shift in fuel utilization characterized by an increased preference for carbohydrate oxidation at the expense of lipid oxidation which may explain why successful weight reduction is so hard to achieve [140]. MacLean et al. [142] believe that these compensatory metabolic adjustments are part of an interrelated group of adaptations in the homeostatic feedback loop between the periphery and the central nervous system that controls body weight. It seems that the homeostatic feedback system defending body weight and adiposity is fundamental to the metabolic drive to regain lost weight [142, 143]. The positive aspect is that modification of this biological predisposition is possible. Interestingly, exercise training seems to positively alter this propensity and has been shown to be important to successful weight maintenance after weight loss programs [144, 145]. Levin et al. [146, 147] reported that regular physical activity lowers the defended level of weight gain and adiposity without compensatory increase in intake and with a favourable alteration in the development of the hypothalamic pathways controlling energy homeostasis as compared to calorically restricted rats. These authors suggested that exercise produces a different set of regulatory signals from caloric restriction that resets the homeostatic balance between energy intake and expenditure toward defence of a lower level of weight gain and adiposity. 

Body weight and fat mass gain and regain following weight loss may be even more critical after menopause since physiological withdrawal of ovarian hormones, by itself, negatively affects the energy balance. Similarly to the above discussion on weight regain, Nicklas et al. [148] suggested that the poor success rate of food restriction treatment in postmenopausal women may be due in part to metabolic adaptations that occur in response to a long period of negative energy balance such as declined fat oxidation, resting metabolic rate, and adipocyte lipolytic responsiveness which predispose the regain of body weight. These authors showed that the addition of endurance exercise to diet-induced weight loss program minimizes these negative metabolic adaptations in postmenopausal women. Similarly, substituting a walking training to a very-low-energy diet in premenopausal obese women improved maintenance of losses in weight and waist circumference and prevented further clustering of metabolic risk factors [149]. As mentioned above, data on Ovx rats suggest that changing from a food restriction regimen to an exercise training program can be an interesting strategy to promote long-term weight reduction in postmenopausal women [135].

## 9. Conclusion

It is becoming increasingly clear that once women reach menopause in their life they are exposed to increasing risks of developing complications due to a decrease in estrogens-related protective effects. Among the protective effects of estrogens, liver fat accumulation seems to be of primary importance due to its important role in the development of insulin resistance, atherosclerosis, and cardiovascular diseases. Information relative to cellular and molecular mechanisms using the animal model indicates that gene expressions of molecules such as SREBP-1c, ChREBP, and SCD-1 involved in the lipogenesis pathway are upgraded with estrogens withdrawal, while molecular markers of the oxidative pathway including CPT-1 and PGC-1 and molecular markers of VLDL production such as MTP and DGAT-2 are reduced with estrogens loss [53, 122]. These observations point to the direction that a reduction in estrogens production results in central effects such as an increase in food intake and a reduction in energy expenditure, but may also metabolically affect tissues such as the liver thus resulting in ectopic fat accumulation. In a recent review paper on NAFLD in older women [21], it was concluded that there is no effective pharmacological treatment available and that lifestyle modifications with weight loss and exercise are regarded as first line treatment. Although there is a lack of information on the role of exercise on liver lipid infiltration during the premenopausal to postmenopausal transition, data on the animal model clearly indicate that exercise training exerts a powerful action in reducing liver fat accumulation especially if exercise training is conducted at the same time as estrogens withdrawals [102, 120]. Exercise training seems to exert an estrogenic-like effect not only on expression of genes involved in lipid accumulation but also on expression of genes of subclinical inflammation in liver [122]. Taken together, it seems that if there is a time in women's life where physical exercise is important, it is with menopause.

## Figures and Tables

**Table 1:** Summary of the central and intrahepatic effects resulting in liver fat accumulation with estrogens withdrawal.

Central effects	Intra-hepatic effects
CNS/hypothalamic effects	Lipid uptake
(i) ↑ Food consumption (ii)↑ Leptin secretion (iii) ↓ Activity and energy expenditure	(i) Unknown (possible mechanism of upregulation of fatty acid uptake via estrogens-dependent pathways, yet to be explored)

Lipid profile and adipose tissue effects	Lipogenesis
(i) Absence of estrogens causes fat redistribution/gain particularly increased intra-abdominal fat and altered lipid homeostasis (portal/fatty acid flux theory)	(i) ↑ SREBP-1c and PGC1*α* (ii) ↑ SCD-1 (iii) ↑ FAS (iv) ↑ ACC (v) ↑ PPAR-*γ*
	Lipid oxidation
	(i) ↓ PPAR-*α* (ii) ↓ HSL (iii) ↓ Fatty acid *β*-oxidation
	VLDL-TG production and secretion system
	(i) ↓ VLDL-TG production in Ovx rats (ii) ↓ MTP and DGAT2

CNS: central nervous system; SREBP-1c: sterol-regulatory-element-binding-protein 1c; PGC1*α*: peroxisome proliferator-activated receptor gamma coactivator-1 alpha; SCD-1: stearoyl-CoA desaturase-1; FAS: fatty acid synthase; ACC: acetyl-CoA carboxylase; PPAR-*α*, -*γ*: peroxysome proliferator-activated receptor-alpha, -gamma; HSL: hormone-sensitive lipase; VLDL-TG: very low density lipoprotein-triglyceride; MTP: microsomal triglyceride transfer protein; DGAT2: diacyl-glycerol acyltransferase-2.
